# In-depth mapping of DNA-PKcs signaling uncovers noncanonical features of its kinase specificity

**DOI:** 10.1016/j.jbc.2024.107513

**Published:** 2024-06-28

**Authors:** Shannon Marshall, Marcos V.A.S. Navarro, Carolline F.R. Ascenҫão, Diego Dibitetto, Marcus B. Smolka

**Affiliations:** 1 Weill Institute for Cell and Molecular Biology, Department of Molecular Biology and Genetics, Cornell University, Ithaca, New York, USA; 2 IFSC Institute of Physics of São Carlos, University of São Paulo, São Carlos, São Paulo, Brazil; 3 Department of Experimental Oncology, Istituto di Ricerche Farmacologiche Mario Negri IRCCS, Milan, Italy

**Keywords:** DNA-dependent serine/threonine protein kinase (DNA-PK), phosphoproteomics, DNA damage response, ionizing radiation, cellular signaling, phosphorylation motif, kinase interplay, kinase specificity

## Abstract

DNA-PKcs is a DNA damage sensor kinase with established roles in DNA double-strand break repair *via* nonhomologous end joining. Recent studies have revealed additional roles of DNA-PKcs in the regulation of transcription, translation, and DNA replication. However, the substrates through which DNA-PKcs regulates these processes remain largely undefined. Here, we utilized quantitative phosphoproteomics to generate a high coverage map of DNA-PKcs signaling in response to ionizing radiation and mapped its interplay with the ATM kinase. Beyond the detection of the canonical S/T-Q phosphorylation motif, we uncovered a noncanonical mode of DNA-PKcs signaling targeting S/T-ψ-D/E motifs. Sequence and structural analyses of the DNA-PKcs substrate recognition pocket revealed unique features compared to closely related phosphatidylinositol 3-kinase-related kinases that may explain its broader substrate preference. These findings expand the repertoire of DNA-PKcs and ATM substrates while establishing a novel preferential phosphorylation motif for DNA-PKcs.

DNA-dependent protein kinase (DNA-PK) is a phosphatidylinositol 3-kinase-related kinase (PIKK) with key roles in the repair of DNA double strand breaks (DSBs) through nonhomologous end joining (NHEJ) (1). It is a holoenzyme composed of a large catalytic subunit, DNA-PKcs, and the Ku70/Ku80 heterodimer (2, 3). DNA-PK is rapidly recruited to broken DNA ends by Ku, which induces conformational changes that activate the catalytic activity of DNA-PKcs (4, 5). While active DNA-PKcs is reported to phosphorylate hundreds of proteins, including several components of the NHEJ complex such as Ku, XRCC4, and XLF (6), the best characterized substrate currently demonstrated to be required for NHEJ is DNA-PKcs itself (7, 8). DNA-PKcs autophosphorylation alters the conformational state of DNA-PKcs engaged at DNA ends, granting access of broken DNA ends to processing enzymes like the nuclease Artemis and the Pol X family polymerases, in addition to stimulating the release of DNA-PKcs from DNA ends (9, 10). Paradoxically, autophosphorylation seems to also limit the extent to which DNA ends can be processed (11). Loss of DNA-PKcs results in severe DNA repair defects, which manifest at the organismal level as severe combined immunodeficiency since V(D)J and class switch recombination require DSB end processing and joining *via* NHEJ (10, 12, 13).

Apart from its canonical role in DNA repair, DNA-PKcs has also been implicated in a range of other nuclear processes. For example, DNA-PKcs promotes faithful replication of telomeres and telomere capping through phosphorylation of hnRNPA1 (14, 15, 16). Additionally, DNA-PKcs has been shown to phosphorylate and regulate various transcription factors. In fact, one of its first identified substrates was the transcription factor SP1 during the formation of promoter-bound transcriptional complexes (17). Inhibition or depletion of DNA-PKcs was reported to result in reduction in RNA Pol II–mediated transcription in a manner that is dependent on the DNA-PKcs substrate TRIM28 (18). The catalytic activity of DNA-PKcs is also relevant for ribosome biogenesis. DNA-PKcs makes Ku-dependent contacts with the snoRNA in the U3 component of the ribosomal small subunit processome, where it is activated and autophosphorylated (19). Mutations in DNA-PKcs that impair its catalytic activity or block autophosphorylation halt rRNA processing, resulting in ribosome deficiency and translation defects in hematopoietic cells. More recently, DNA-PKcs has been shown to be involved in the control of DNA replication in response to replication stress by promoting replication fork reversal and the slow-down of fork progression (20).

The precise targets through which DNA-PKcs mediates its functions in NHEJ-mediated DNA repair, RNA processing and DNA replication remain largely unknown. Identification of functional substrates is complicated by the lack of a complete map of DNA-PKcs signaling and by the partial redundancy of its roles and substrates with the ATM kinase. Here, we performed phosphoproteomic analysis of DNA-PKcs signaling and mapped its division of labor with the ATM kinase. To gain deep coverage of the DNA-PKcs and ATM signaling network, we used Lig4 deficient mouse Pre-B cells that are unable to efficiently repair DSBs, and therefore hyperaccumulate DNA-PKcs and ATM signaling induced by ionizing radiation (IR). Our experimental setup allowed us to define the unique contributions of ATM and DNA-PKcs kinases and uncover a novel S/T-ψ-D/E motif preferentially targeted by DNA-PKcs. These findings expand the list of DNA-PKcs and ATM substrates and establish a novel preferential phosphorylation motif for DNA-PKcs.

## Results

### In-depth mapping of the signaling response induced by IR in mouse Pre-B cells

**Figure 1:**
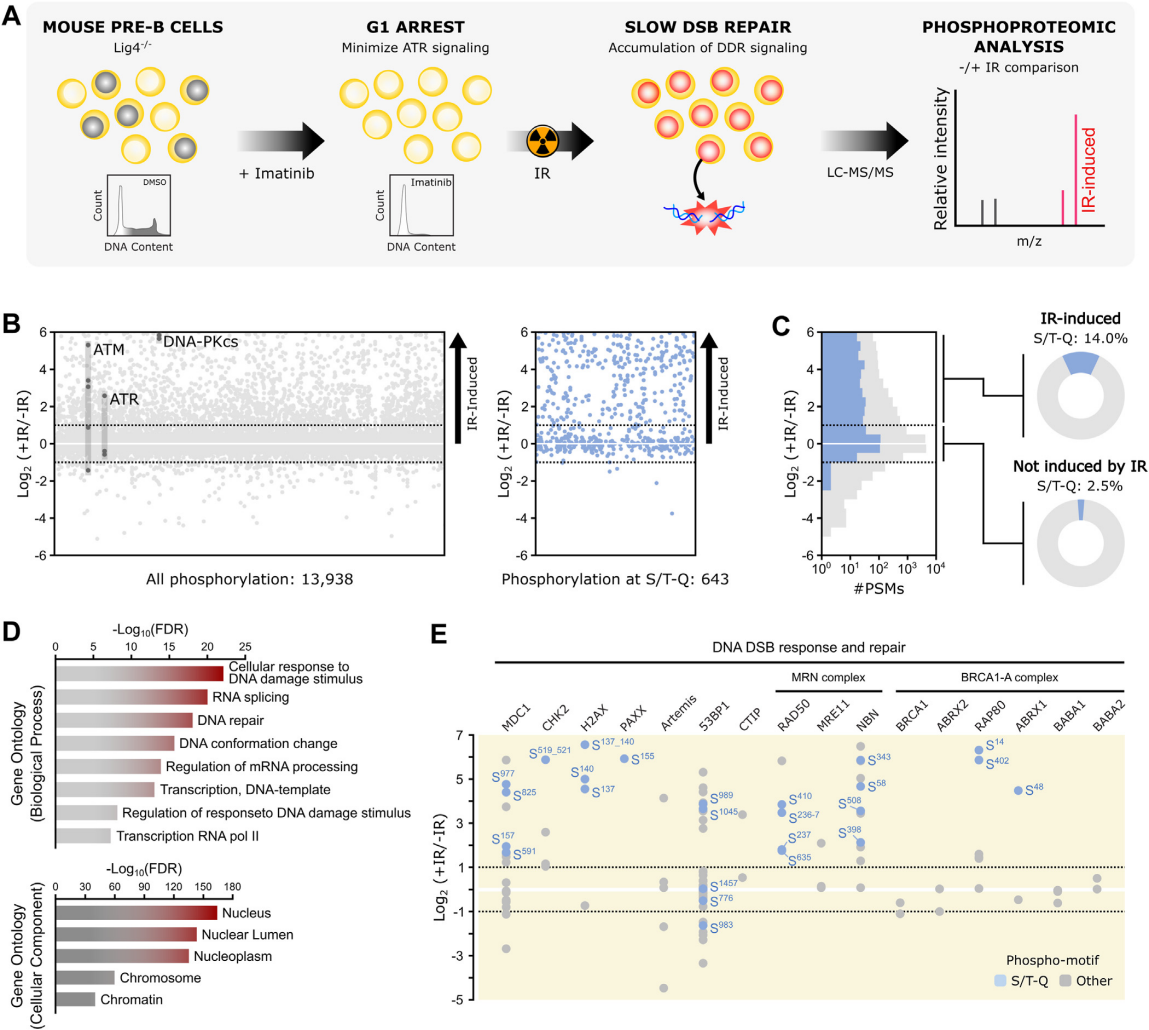
**In-depth phosphoproteomic analysis of the cellular response to IR in mouse Pre-B cells.***A*, workflow for maximizing the detection of IR-induced phosphorylation in mouse Pre-B cells. Pre-B cells have a high nuclear to cytosolic protein ratio, which facilitates detection of low abundance nuclear signaling events. Deletion of LIG4 prevents the rapid repair of IR-induced breaks, leading to accumulation of IR-induced signaling events. Pre-B cells from Lig4^−/−^ mice were first arrested in G1 with imatinib. Following arrest, cells were irradiated with 20 Gy IR and harvested for quantitative phosphoproteome analysis. Quantification of phosphoproteomics changes was accomplished using stable isotope labeling of amino acids in cell culture. *B*, the plot on the *left* compares the phosphoproteome of Pre-B cells treated with IR to the phosphoproteome of control (untreated) cells. The plot on the *right* displays only peptides phosphorylated at the S/T-Q motif. *Dashed lines* indicate a 2-fold change in abundance. Phosphorylation sites detected in ATR, ATM, and DNA-PKcs are highlighted. Each point represents the average of at least two independent experiments (see Fig. S1). *C*, cumulative plot of S/T-Q sites (*blue*) *versus* all sites (*gray*). *Pie charts* on the *left* highlight the proportion of S/T-Q sites not induced (−1 < Log_2_ (+IR/-R) < 1) and induced (Log_2_ (+IR/-R) > 1) by IR. *D*, curated gene ontology analysis showing enriched gene ontology terms (biological processes and cellular components) among IR-induced phosphorylation sites. *E*, selected phosphorylation sites identified in proteins involved in DNA double strand break response and repair. *Dashed lines* indicate a 2-fold change in abundance. S/T-Q sites are *light blue*. IR, ionizing radiation; LIG4, ligase IV.

We conducted a phosphoproteomic analysis of IR-induced phosphorylation using mouse Pre-B cells transformed with Abelson murine leukemia virus and deficient for DNA ligase IV (LIG4) (21). This system provides several advantages for mapping DNA-PKcs signaling with high coverage (Fig. 1*A*). First, these Pre-B cells can be robustly arrested in G1 using treatment with the ABL kinase inhibitor imatinib, helping to minimize confounding effects from ATR activation in S-G2 (22, 23). Second, Abelson Pre-B cell nuclei account for approximately 70% of their cellular volume (24), which enhances the coverage of nuclear proteins in our phosphoproteomics analysis. Third, we used cells deficient for LIG4, a critical enzyme for the repair of DSBs through NHEJ, which is the predominant repair mechanism during the G1 phase. Lig4^−/−^ cells accumulate unrepaired breaks and persistent DSB signaling (25).

G1-arrested cells were treated with 20 Gy of IR and then harvested 90 min later for analysis using stable isotope labeling of amino acids in cell culture (SILAC)-based quantitative mass spectrometry. Over 16,000 phosphorylation sites were identified, mapping to over 3900 proteins (Table S1). To ensure confidence in our dataset, we utilized a bowtie filtering approach based on reciprocal labeling, which has been shown to mitigate false positive identifications and enhance quantification of phosphopeptides with low number of spectral matches in phosphoproteomic analyses (26). Following this rigorous filtering process, a total of 13,938 high-quality phosphopeptides were identified and quantified (Figs. 1*B* and S1). Among these, 2258 exhibited at least a 2-fold increase in abundance after IR treatment and were categorized as IR-induced. To our knowledge, this represents the most comprehensive set of IR-induced phosphorylation events reported in mammalian cells (27, 28, 29, 30, 31, 32). As expected, the preferential phosphorylation motif for ATM and DNA-PKcs, phosphoS/T-Q, was enriched in the set of IR-induced sites (Fig. 1, *B* and *C*). Moreover, we detected phosphorylation sites in ATM and DNA-PKcs that were highly induced by IR (Fig. 1*B*), consistent with the expectation that these two PIKKs are engaged in the IR-induced response in G1-arrested Pre-B cells. Gene ontology analysis indicated that the set of phosphorylation events induced by IR was enriched for proteins involved in DNA damage response and repair, as well as mRNA-related processes, with nuclear and chromatin proteins being highly represented (Fig. 1*D*). Proteins with established roles in the DNA DSB response displayed several IR-induced phosphorylation events, with a predominance of S/T-Q phosphorylation sites, but with other non-S/T-Q sites also detected (Fig. 1*E*). Taken together, these results highlight the effectiveness of our approach in providing a comprehensive dataset of IR-induced phosphorylation. Since the Pre-B cells used are efficiently arrested in G1, this dataset is likely to be overrepresenting targets of DNA-PKcs and ATM and limiting the contributions of ATR to the generated signaling responses.

### Mapping ATM and DNA-PKcs–dependent phosphorylation following IR

**Figure 2:**
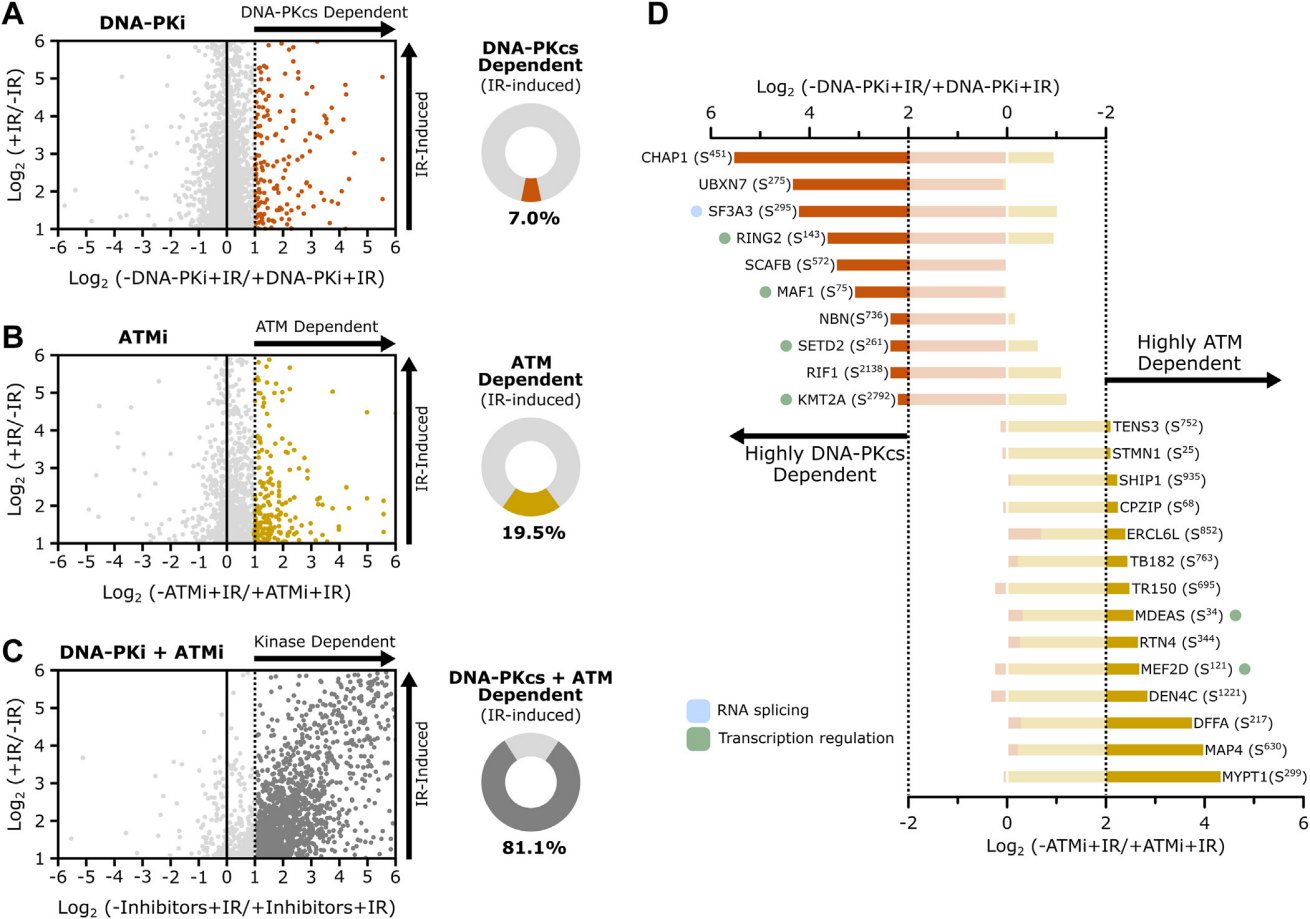
**Mapping ATM- and DNA-PKcs–dependent signaling following IR treatment.***A*–*C*, *scatter plots* comparing the phosphoproteomes of IR-treated mouse Pre-B cells in the absence (*y*-axis) and presence (*x*-axis) of standalone DNA-PKi (*A*) or ATMi (*B*), and combined kinase inhibitors (*C*). The *pie charts* to the *right* of the *scatter plots* illustrate the proportion of IR-induced phosphorylation sites dependent on DNA-PKcs and ATM catalytic activities. *D*, phosphorylation sites highly dependent on either DNA-PKcs (*orange*) or ATM (*yellow*). *Dashed lines* indicate 4-fold abundance change. Proteins involved in RNA splicing and transcription regulation are highlighted in *light blue* and *lightgreen*, respectively. ATMi, ATM inhibitor; DNA-PKi, DNA-PKcs inhibitor; IR, ionizing radiation.

The redundancy among PIKKs in the response to DNA damage presents a major challenge in pinpointing specific kinase targets within DDR signaling pathways (33, 34). Leveraging our strategy to effectively minimize ATR signaling, we examined the individual and combined contributions of ATM and DNA-PKcs to the overall IR-induced signaling through selective kinase inactivation using the DNA-PKcs inhibitor (DNA-PKi) NU7441 and the ATM inhibitor (ATMi) KU55933 (Table S1) (35, 36, 37, 38). Inhibition of DNA-PKcs impaired 145 sites, which represents ∼7% of all the IR-induced phosphorylation sites detected in this analysis (Fig. 2*A*). Individual inhibition of ATM impaired 185 sites, which represents ∼19.5% of all the IR-induced phosphorylation sites detected in this specific analysis (Fig. 2*B*). Simultaneous inhibition of DNA-PKcs and ATM caused a reduction in 929 sites, which represents ∼81.1% of the IR-induced phosphorylation sites detected in the experiment in Figure 2*C*. These results underscore the redundancy of these kinases, where one kinase can largely compensate for the loss of activity of the other kinase. The drastic reduction of IR-induced phosphorylation in the absence of ATM and DNA-PKcs activity is consistent with the prevailing view that these kinases are the major responders to IR-induced DNA damage in G1. About 90% of peptides phosphorylated at the preferred S/T-Q consensus motif were reduced upon dual inhibition of ATM and DNA-PKcs (Fig. S2).

Despite the extensive overlap between ATM and DNA-PKcs–dependent substrates in the IR-induced response, our data revealed phosphorylation sites predominantly dependent on each of these kinases, implying a degree of substrate specificity unique for ATM and DNA-PKcs (Fig. 2*D*). For instance, while the phosphorylation status of a range of proteins involved in RNA biology is influenced by both ATM and DNA-PKcs (Fig. S3), phosphorylation of SF3A3 S^295^—a core component of the U2 small nuclear ribonucleoprotein (39)—is highly dependent on DNA-PKcs but only mildly dependent on ATM (Fig. 2*D*). Furthermore, our data reveals differential targeting of factors involved in transcription regulation, with the phosphorylation of factors like RING2 and SETD2 being highly dependent only on DNA-PKcs, and MDEAS and MEF2D proteins undergoing phosphorylation only in an ATM-dependent manner (Fig. 2*D*). Overall, our data reveal the division of labor for ATM and DNA-PKcs kinases, allowing the delineation of IR-induced signaling into distinct groups based on their level of dependency toward each or both kinases.

### Identification and validation of a novel preferential motif for DNA-PKcs phosphorylation

**Figure 3:**
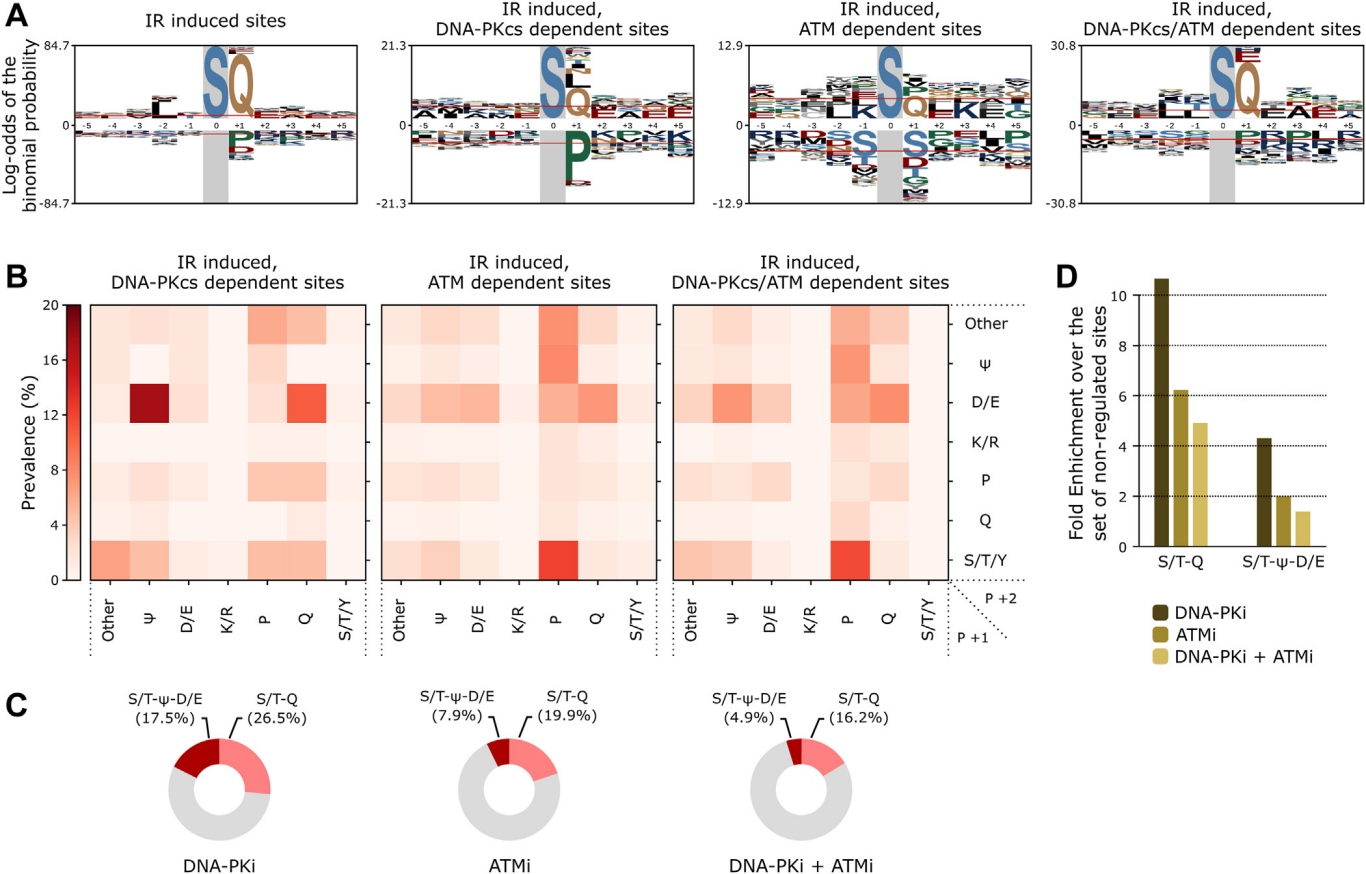
**Motif analysis of IR-induced and kinase-dependent phosphorylation sites.***A*, sequence logo analysis of indicated sets of phosphorylation sites induced by IR (Log_2_ ratio of +IR/-IR is > 1). Phosphorylation sites dependent on the indicated kinases have Log_2_ ratio (-Inhibitors+IR/+inhibitors+IR) > 1. Plots represent the Log-odds of the binomial probability with Bonferroni correction (40). Serine occurrence at the phosphorylation site exceeded 80%, and this position was fixed in the sequence logos. *B*, heat maps showing the prevalence of the indicated amino acid groups at the +1 (*horizontal*) and +2 (*vertical*) positions within the highlighted set of IR-induced, kinase-dependent phosphorylation sites, compared to nonregulated sites (See Fig. S4*B*). IR-induced and kinase-dependency were defined as in *A*. *C*, *pie charts* depicting the relative proportions of S/T-Q and S/T-ψ-D/E motifs induced by IR and dependent on the indicated kinase(s). *D*, *bar chart* illustrating the enrichment of S/T-Q and S/T-ψ-D/E motifs in each of the indicated kinase-dependent groups over the set of nonregulated sites. For each motif, the fold enrichment represents their prevalence in the group of regulated phosphorylation sites divided by their prevalence in the nonregulated set (See Fig. S4, *B* and *C*). IR, ionizing radiation.

In search of sequence motifs specific to ATM or DNA-PKcs signaling, we investigated the amino acid preferences within phosphorylation motifs across our datasets. Sequence probability logos revealed a strong enrichment of glutamine at the +1 position of phosphorylation events induced by IR (Fig. 3*A*) (40). This aligns with the established S/T-Q motif preference of PIKK kinases (34). Sequence analysis of sites induced by IR and inhibited by dual DNA-PKi and ATM inhibitor treatment showed similar enrichments for the position +1 (Fig. 3*A*). Notably, the logo profile for sites impaired by monotreatment with DNA-PKcs inhibitor displayed a marked enrichment in bulky hydrophobic residues at the +1 position, mainly L, and I to a lesser extent, as well as enrichment in asparagine (Fig. 3*A*). Given the apparent coenrichment of residues L/I at +1 with glutamic acid at +2 (Fig. S4*A*), we performed a motif prevalence analysis combining these positions (Fig. 3, *B* and *C* and Fig. S4, *B* and *C*). For these analyses, we also grouped V and F together with I and L, given their similar bulky hydrophobic (ψ) property and their prevalence in the group of DNA-PKcs–dependent and IR-induced sites (Table S1). As shown in Figure 3, *B*–*D*, the set of IR-induced phosphorylation sites dependent on DNA-PKcs exhibited a high prevalence and enrichment of motifs with bulky hydrophobic (ψ - F, I, L, or V) and acidic (D/E) residues at positions +1 and +2, respectively. While this S/T-ψ-D/E motif was marginally enriched in ATM-dependent sites, it accounted for 17.5% of the DNA-PKcs–dependent sites, reflecting an almost 4.5-fold increase over its frequency in nonregulated phosphorylation events (Fig. 3*D*). We note that, given the relatively high prevalence of the S/T-Q-D/E motif in the set of DNA-PKcs–dependent sites (Fig. 3*B*), D/E at +2 position may represent a general preference for DNA-PKcs independently of the amino acid at the +1 position.

**Figure 4:**
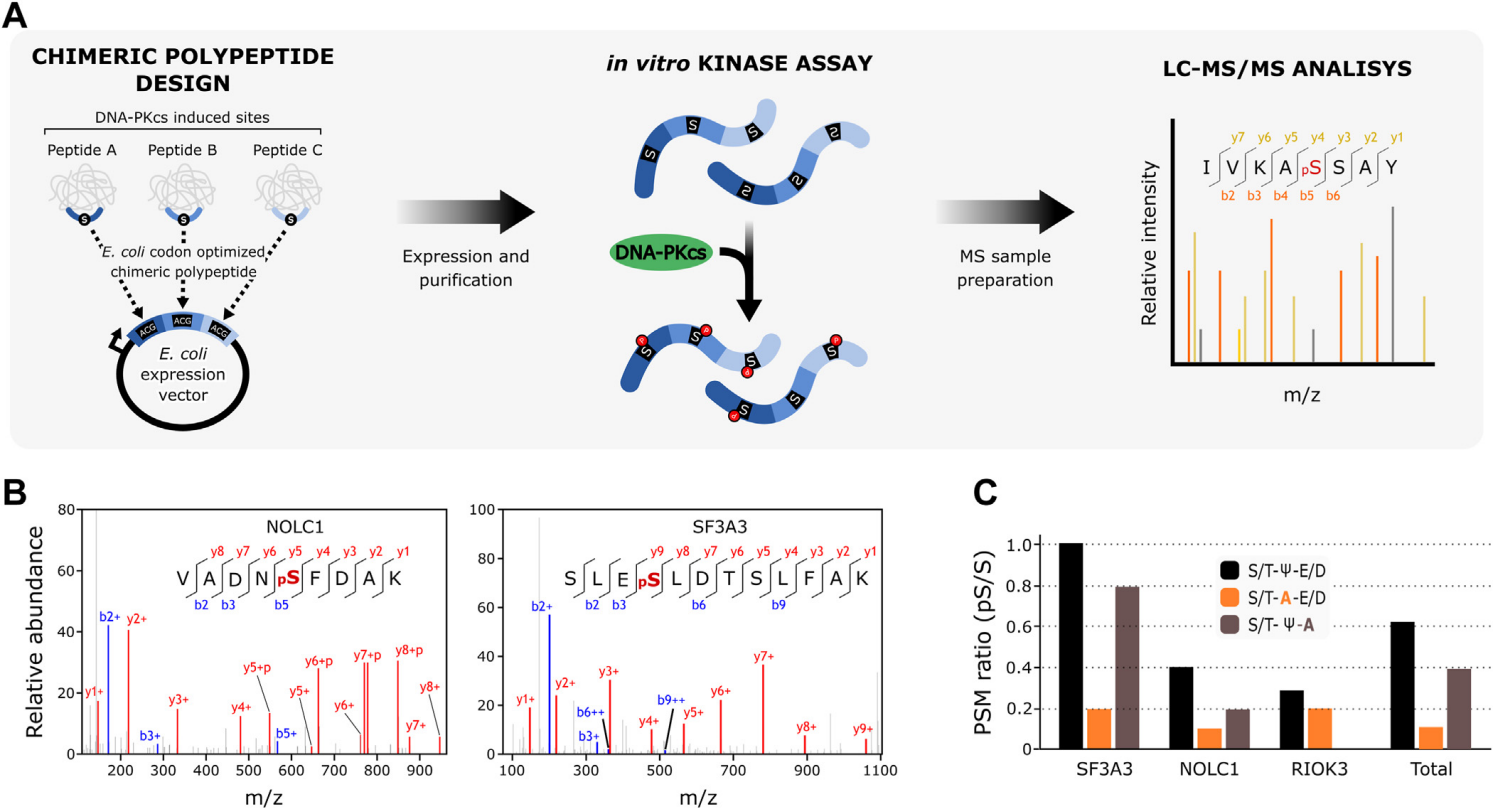
**DNA-PK *in vitro* kinase assay validates phospho-targets as direct substrates.***A*, schematic workflow of the *in vitro* kinase assay. *B*, representative MS2 spectra confirming the identification of the phosphorylated peptides derived from NOLC1 and SF3A3, encoded within the synthetic chimeric peptide. *C*, *bar chart* depicting the ratio of number of PSMs for phosphopeptides divided by the number of PSMs of their unphosphorylated counterparts (pS/S) identified in the mass spectrometry experiments (Fig. S5). S/T-A-E/D and S/T-ψ-A indicate alanine substitutions at the +1 or +2 positions. PSM, peptide spectral match.

To determine whether the S/T-ψ-D/E motif is directly targeted by DNA-PKcs, we used an *in vitro* assay with the purified kinase domain of DNA-PKcs, commercially available, and a chimeric polypeptide substrate designed with sequences of peptides containing the S/T-ψ-D/E motif, representing detected DNA-PKcs–dependent phosphorylation events (Fig. 4*A*). We selected seven coding regions for 11-residue peptides harboring S/T-ψ-D/E motifs at the central position (Fig. S5) and cloned them in tandem into an *Escherichia coli* expression vector. Following expression and purification, this chimeric substrate was incubated with active recombinant DNA-PKcs, and the resulting phosphopeptides were detected using LC-MS/MS. While peptide spectral matches provide a crude measure of phosphorylation extent in this qualitative assay, our results show that DNA-PKcs can directly phosphorylate all of the selected peptides at the S/T-ψ-D/E motif (Figs. 4*B* and S5). Notably, mutating the bulky hydrophobic residue in the +1 position or the negative residue in the +2 position to an alanine reduced phosphorylation events, with a more pronounced effect when mutating position +1 (ψ to A mutation) (Figs. 4*C* and S5). This is consistent with the S/T-ψ-D/E motif being a preferential and specific motif for DNA-PKcs phosphorylation, although the inherent sensitivity of mass spectrometry should be considered when interpreting these *in vitro* findings.

### Sequence and structural comparative analyses reveal unique features of the DNA-PKcs substrate recognition pocket

**Figure 5:**
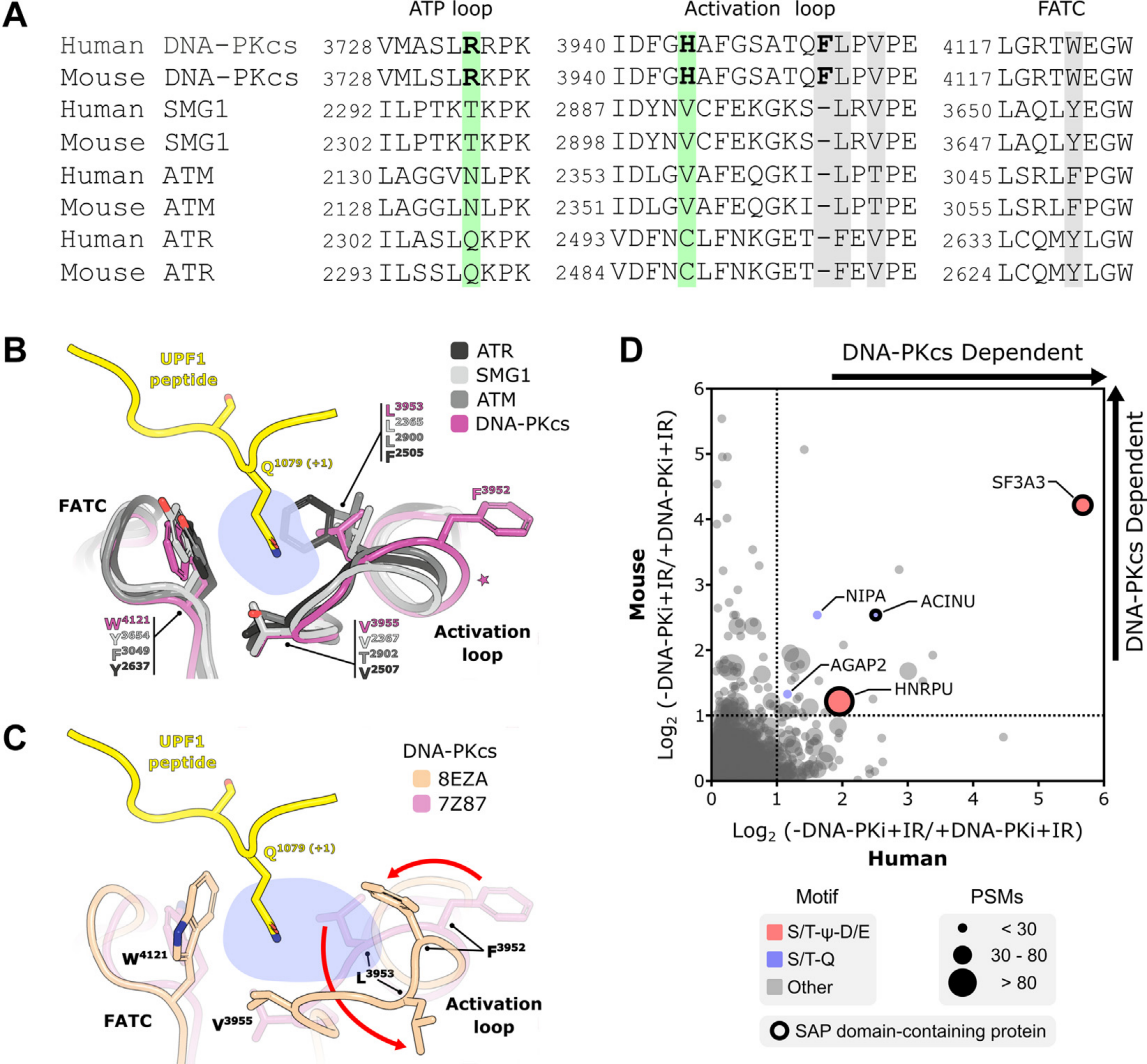
**Unique sequence and structural features of the DNA-PKcs substrate recognition pocket potentially underlying the preferential phosphorylation of S/T-ψ-D/E motifs.***A*, sequence alignment of kinases from the PIKK family from mouse and human highlighting the FATC domain, ATP loop, and activation loop. Key residues potentially involved in the recognition of residues at position +1 and +2 of peptide substrates are highlighted in *gray and green*, respectively (41, 42). *B*, structural superimposition of DNA-PKcs (PDB ID 7Z87), ATR (PDB ID 5YZ0), ATM (PDB ID 8OXO), and SMG1 (PDB ID 6Z3R) showing the recognition pocket (*shaded blue area*) with Gln at the position +1 of the UPF1 peptide in complex with SMG1. Conserved residues involved in the formation of this hydrophobic cage are indicated by *black round arrows*. A *star* highlights the relatively longer segment in the DNA-PKcs activation loop caused by the conserved F^3952^ insertion. *C*, superposition of two DNA-PKcs structures (PDB IDs 8EZA and 7Z87) showing the structural flexibility of the region involved in the recognition of residues at the position +1 of peptide substrates. DNA-PKcs structures are viewed in the same orientation as in *panel B*. The *shaded blue area* highlights the modified position +1 recognition pocket generated by the structural rearrangement of residues L^3953^ and F^3952^ (*red arrows*). The UPF1 peptide bound to superimposed SGM1 structure (PDB ID 6Z3R) is shown in *yellow*. *D*, *scatter plot* comparing DNA-PKcs dependency of IR-induced phosphorylation sites found in both Pre-B cells (mouse) and HCT116 cells (human). S/T-Q and S/T-ψ-D/E motifs are depicted in *blue* and *red*, respectively. The number of PSMs represents the combined count from both human and mouse phosphoproteomes. SAP domain–containing proteins are highlighted with a *black circle*. FATC, C-terminal FRAP, ATM, TTRAP; PIKK, phosphatidylinositol 3-kinase-related kinase; PSM, peptide spectral match; SAP, SAF-A/B, acinus and PIAS.

To gain insights into the molecular basis for the relaxed specificity of DNA-PKcs for the +1 position in substrates, we compared the sequence and structure of the DNA-PKcs catalytic domain (including the ATP loop, activation loop, and the C-terminal FRAP, ATM, TTRAP domain) to other kinases from the PIKK family (Fig. 5, *A*–*C*). Sequence alignment of mouse and human PIKKs demonstrated that DNA-PKcs contains an extra residue within the highly conserved activation loop (Fig. 5*A*). We superimposed the structures of the substrate recognition site of these four kinases, modeling over structures of ATM and SMG1 bound to substrate peptides where a conserved hydrophobic cage accommodates the glutamine residue at the +1 position of the substrate (41, 42) (Fig. 5*B*). Interestingly, while DNA-PKcs adopts this pocket with alignment of its W^4121^, L^3953^, and V^3555^ residues, we noticed that reported alternative structures show marked differences, suggesting that the pocket of DNA-PKcs can adopt multiple configurations (Fig. 5*C*). This is consistent with the unique additional residue (F^3952^) in DNA-PKcs’s activation loop and suggests that its hydrophobic cage may have increased flexibility, allowing the recognition of bulky hydrophobic residues at the +1 position of substrates (Fig. 5, *A*–*C*).

These sequence and structural analyses show that both mouse and human DNA-PKcs share distinct features in their catalytic domain that differentiate them from other PIKKs and suggest that human DNA-PKcs may also be able to target S/T-ψ-D/E motifs. To test this possibility, we carried out a phosphoproteomics analysis of IR-treated asynchronous human HCT116 cells, comparing cells mock-treated or treated with DNA-PKcs inhibitor. Using a bowtie filter analysis analogous to our prior mouse Pre-B cell experiment, we identified 11,438 phosphorylation sites, with 274 showing at least a 2-fold reduction upon DNA-PKcs inhibition (Fig. S6, *A* and *B* and Table S2). Surprisingly, enrichment analysis of DNA-PKcs–dependent sites revealed only a minor 2-fold increase of canonical PIKK S/T-Q motifs and no enrichment of S/T-ψ-D/E motifs (Fig. S6, *C*–*E*). Functional enrichment analysis on the list of DNA-PKcs–dependent sites did not reveal gene ontology terms related to DNA repair and DNA damage responses (Fig. S6*F*). We attribute this, in part, to the lower coverage of the human phosphoproteome and a higher accumulation of pleiotropic events downstream of DNA-PKcs signaling than the phosphorylation events directly mediated by DNA-PKcs. These results using a human cell line also highlight the benefits of the mouse Pre-B cell system for mapping DNA-PKcs signaling and characterizing its sequence specificity (Fig. 1*A*). Despite the experimental limitations of the phosphoproteomic analysis in HCT116 cells, we asked whether conserved DNA-PKcs signaling events between mouse and human could be detected at the same S/T-ψ-D/E motifs of the proteins identified to be targeted by DNA-PKcs in mouse Pre-B cells. To identify conserved signaling events under DNA-PKcs regulation, we compared the HCT116 dataset with the mouse Pre-B cell dataset using the DIOPT Ortholog Finder (43). Homologous proteins between the human and mouse datasets were aligned and we then searched for conserved phosphorylation sites. This analysis unveiled over 1200 conserved phosphorylation sites in mouse Pre-B and human HCT116 cells (Table S3), where 31 sites (28 unique proteins) were consistently DNA-PKcs–dependent in both species (Fig. 5*D*), including three sites phosphorylated at the canonical S/T-Q motif and two sites phosphorylated at the S/T-ψ-D/E motif. Among the proteins found to be targeted by DNA-PKcs in both mouse and human cells were kinases and phosphatases (Fig. S7), consistent with the identification of DNA-PKcs–dependent phosphorylation at motifs other than S/T-Q or S/T-ψ-D/E, which could represent events controlled by kinases targeted by DNA-PKcs. Notably, several proteins containing DNA-PKcs–dependent phosphorylation at S/T-Q or S/T-ψ-D/E (potential direct targets) were nucleic acids and chromatin binding proteins (Fig. S7). Of these, three proteins (HNRPU, SF3A3, and ACINU) contained a SAF-A/B, acinus and PIAS domain, which is an RNA/DNA-binding module typically present in proteins involved in DNA repair and RNA biology (44, 45, 46, 47). SF3A3 is one of the proteins we validated to be phosphorylated by DNA-PKcs *in vitro* at an S/T-ψ-D/E motif (S^295^LD). Overall, despite the limitations of the phosphoproteomic analysis in HCT116 cells for mapping DNA-PKcs signaling, these results support the notion that the ability to phosphorylate S/T-ψ-D/E motifs is a conserved feature of DNA-PKcs.

## Discussion

DNA-PKcs plays roles in multiple processes, including DNA repair, replication, translation, and transcription. Despite established biological effects downstream of DNA-PKcs–mediated signaling being reported, the key substrates involved remain largely unknown. Mass spectrometry–based phosphoproteomics has enabled the mapping of signaling orchestrated by PIKK kinases, which has been mostly applied to the study of ATM and ATR signaling. Recent work has begun to map DNA-PKcs–signaling events (48), although the full scope of DNA-PKcs–dependent signaling remains incomplete given intrinsic issues with low coverage in phosphoproteomic analysis of complex protein mixtures. Here, to further expand the map of DNA-PKcs–dependent signaling, we used mouse Pre-B cells lacking the LIG4 ligase, which accumulate unrepaired breaks and amplify DNA damage signaling, enabling increased coverage in the identification of DNA-PKcs signaling. This approach expanded the identification of DNA-PKcs–dependent signaling events and uncovered a novel motif preferentially targeted by DNA-PKcs, which was subsequently corroborated through *in vitro* kinase assays and cross-species analyses with human cells. The discovery of novel conserved features of DNA-PKcs kinase specificity opens new directions to investigate the mechanisms by which this kinase exerts its regulatory roles on DNA repair as well as on a multitude of other processes such as RNA processing and replication fork dynamics.

Our analysis identified over 2000 phosphorylation sites induced by IR in G1 arrested Pre-B cells and revealed that ATM and DNA-PKcs are responsible for the majority of the IR-induced signaling response in G1. Through the application of individual kinase inhibitors, we further revealed specific phosphorylation targets that rely exclusively on DNA-PKcs activity and are independent of ATM. Within this group of substrates, we observed an enrichment of the S/T-ψ-D/E motif in addition to the well-characterized S/T-Q motif. Notably, the ability of DNA-PKcs to phosphorylate S/T-ψ-D/E motifs in HNRPU (S^59^) and XRCC4 (S^260^) has been reported in low-throughput *in vitro* experiments (49, 50, 51). More recently, a large-scale study utilized synthetic peptide libraries to determine the *in vitro* specificity of human kinases, which further corroborates our findings by demonstrating that DNA-PKcs displays a preference for leucine at +1 position and D or E at +2 position within the libraries of amino acids independently fixed at positions surrounding the phospho-acceptor site (52). Our study establishes the prevalence of the S/T-ψ-D/E motif *in vivo* and identifies several novel putative substrates phosphorylated at this motif. Importantly, the ability of DNA-PKcs to accommodate bulkier hydrophobic residues at the +1 position could be linked to unique structural features within its substrate recognition pocket. For instance, while residues in the activation loop of PIKK kinases involved in recognizing glutamine at the position +1 of substrates are conserved in DNA-PKcs, it contains a unique phenylalanine (F^3952^) in this region. This residue insertion may promote structural flexibility, potentially contributing to versatility in substrate recognition (Fig. 5, *A*–*C*). Interestingly, adjacent to DNA-PKcs’ phenylalanine insertion is a unique TQ^3951^ signature, whose phosphorylation has been shown to be critical for DNA-PKcs function (53). In the future, it will be interesting to investigate whether this phosphorylation event induces conformational changes associated of DNA-PKcs's substrate recognition. This potential interplay between phosphorylation-induced regulation and substrate specificity offers an interesting avenue to explore the control of DNA-PKcs regulation. It also raises the possibility that the preferential phosphorylation motif for DNA-PKcs may be context-dependent, switching based on specific cellular processes such as DNA repair or RNA-related functions.

In conclusion, our findings uncover novel conserved features of DNA-PKcs kinase specificity. Further exploration of these noncanonical modes of DNA-PKcs signaling holds the potential to address outstanding questions, such as DNA-PKcs’s roles in regulating DNA repair–independent processes such as its role in the control of RNA associated processes and replication fork dynamics. The importance of comprehensively understanding DNA-PKcs signaling is further underscored by the use of DNA-PKcs inhibitors in clinical trials for cancer therapy (54). For instance, our recent discovery that DNA-PKcs catalytic activity is essential for fork reversal expands the potential applications of DNA-PKcs inhibitors in combination therapies beyond their current use in conjunction with radiotherapy (20). The use of DNA-PKcs inhibitors in rationalized combination therapies is particularly interesting given that these inhibitors are largely innocuous to noncancer cells. Elucidation of novel DNA-PKcs substrates and signaling modalities will continue to be crucial for improving our understanding of DNA-PKcs biology and our ability to design improved therapeutic approaches.

## Experimental procedures

### Mammalian cell culture and cell cycle arrest

Mouse Pre-B cells (Lig4−/−) were a gift from Barry Sleckman and were grown at 37 ⁰C in Dulbecco’s modified Eagle medium (DMEM) supplemented with 10% bovine calf serum, 1% nonessential amino acids, 0.0004% beta mercaptoethanol, and penicillin/streptomycin solution (100 U/ml). Pre-B cells were arrested by treatment with 3 μM imatinib for 48 h at a density of 2 × 10^6^ cells/ml. Cell cycle arrest was confirmed *via* flow cytometry by observing the loss of the populations representing S and G2/M phase cells after staining total DNA content with propidium iodide. Fifty milliliters cultures (100 × 10^6^ Pre-B cells) were used for phosphoproteomic experiments. Human HCT116 cells were purchased from the American Type Culture Collection and grown at 37 °C in DMEM supplemented with 10% BCS, 1% nonessential amino acids, and penicillin/streptomycin solution (100 U/ml). Metabolic labeling was performed by growing cells for a minimum of five population doublings in SILAC medium supplemented with either “light” or “heavy” isotopes of lysine and arginine (Thermo Fisher Scientific).

### Induction of DNA DSBs *via* IR

Cells were exposed to 20 Gy of gamma radiation (0.89 Gy/min) from a Cs-137 source. Cell culture dishes were positioned at a designated location in the center of the chamber to ensure maximum dosage. Dishes were continuously rotated to guarantee even distribution of the radiation across the entire sample. Cells were harvested 90 min after the treatment. In experiments involving kinase inhibition, cells were incubated at 37 °C with 10 μM of ATM (KU-55933) and/or DNA-PKcs (NU7441) inhibitors for 1 h prior to IR treatment.

### Protein expression and purification

For the chimeric polypeptides, 11-residues long sequences encoding ten S/T-ψ-D/E sites (Fig. S5), as identified in our phosphoproteomes, were codon-optimized for *E. coli* protein expression, and arranged back-to-back to create synthetic chimeric peptide oligonucleotides, both WT and positional mutants (S/T-A-D/E and S/T-ψ-A). These synthetic oligonucleotides were synthesized and cloned into a bacterial expression vector based on pET28a (Novagen), which adds a cleavable 6xHIS-SUMO tag at the N terminal (pETSUMO). The chimeric polypeptides were overexpressed in *E. coli* BL21 (DE3). The cultures were grown at a temperature of 37 °C in LB medium and supplemented with 50 μg/ml kanamycin. Upon reaching an absorbance at 600 nm (*A*600) of ∼0.6, protein expression was induced by the addition of 0.3 mM IPTG, and allowed to proceed for 16 h at a temperature of 18 °C. Following this, the cells were collected *via* centrifugation and resuspended in lysis buffer (25 mM Hepes, 300 mM NaCl, 20 mM imidazole, pH 8.0). After lysis through sonication, clear lysates were extracted through centrifugation (20,000*g*), and then loaded onto columns containing 1 ml of pre-equilibrated Co-NTA resin (Malvergen) with lysis buffer. The resin was washed with 10 column volumes (CVs) of lysis buffer and followed by an additional washing step with 10 CV of buffer A (25 mM Hepes, 300 mM NaCl, pH 8.0). Protein elution was then conducted using five CV of buffer B (25 mM Hepes, 300 mM NaCl, 300 mM imidazole, pH 8.0). The 6xHis-SUMO moiety of eluted proteins was removed by specific cleavage using the yeast protease Ulp-1. Finally, the cleaved proteins underwent size-exclusion chromatography using a Superdex 75 column (Cytiva) that was pre-equilibrated with a buffer consisting of 25 mM Hepes at pH 8.0 and 150 mM NaCl. This step was crucial for separating the expressed proteins from the cleaved fusion tags and Ulp-1. Purified proteins were concentrated on Cytiva filters (10 KDa cut-off) to ∼10 mg/ml and stored at −80 °C.

### *In vitro* kinase assay

The assays were conducted using the DNA-PK Kinase Enzyme System (Cat # V4106, Promega), following the provided instructions. Briefly, reaction mixtures were composed of 10 μg of chimeric polypeptide, 0.1 mg/ml bovine serum albumin, 1X activation buffer (10 μg/ml calf thymus DNA), 1 mM DTT, 1X reaction buffer, 50 units of DNA-PK, and 150 μM ATP, in a total volume of 50 μl. The reactions were initiated by adding ATP, followed by incubation at room temperature for 1 h. Once completed, the reactions were subjected to a 5-min incubation at 95 °C to inactivate the DNA-PK, flash-frozen in liquid nitrogen, and preserved at −80 °C.

### Sample preparation for phosphoproteomic analysis

After treatment, Pre-B cells were harvested by centrifugation at 1000*g* for 5 min. HCT116 cells were detached from cell culture plates with 0.25% Trypsin (Thermo Fisher Scientific). Trypsin was deactivated by adding 4X volume of fully supplemented DMEM. Then, the cells were pelleted by centrifugation at 1000*g*. Harvested cell pellets were washed once with cold PBS, followed by lysis with cold modified radioimmunoprecipitation assay buffer (50 mM Tris–HCl, pH 7.5, 150 mM NaCl, 1% Tergitol, and 5 mM EDTA supplemented with complete EDTA-free protease inhibitor cocktail (Roche) and PhosSTOP (Millipore) for 30 min on ice. Four milligrams of light and heavy lysates were mixed then reduced and denatured with 1% SDS and 5 mM DTT, respectively at 42 °C. Cysteines of the denatured proteins were alkylated by incubation with 25 mM iodoacetamide for 15 min in the dark. Lysates were then precipitated with a cold precipitation solution (50% acetone. 49.9% ethanol, 0.1% acetic acid). After precipitation, the protein pellet was solubilized with 2 M urea then digested with TPCK-trypsin overnight at 37 ⁰C. Digested peptides were then desalted using a Waters 20 mg Sep-pak C18 column then dried in a SpeedVac and resuspended in 1% acetic acid. Phosphopeptides were enriched using High Select Fe-NTA phosphopeptide enrichment kit (Thermo Fisher Scientific). Enriched phosphopeptides were then dried and resuspended in 15 ul water and 10 μl 10% formic acid.

### Hydrophilic interaction liquid chromatography fractionation

Immediately before injection 60 μl of HPLC grade ACN was added to the phosphopeptide samples. Samples were then fractionated *via* hydrophilic interaction liquid chromatography. A gradient was generated using three buffers: Buffer A (90% ACN), Buffer B (75% ACN and 0.0005% TFA), and Buffer C (0.025% TFA). The gradient consisted of 100% Buffer A at 0 min, 88% Buffer B and 12% Buffer C at 5 min, 60% Buffer B and 40% Buffer C at 30 min, and 5% Buffer B and 95% Buffer C from 35 to 40 min. Fractions were collected every 60 s between minutes 8 and 34. Fractions were then dried in a speedvac and resuspended in 0.1% TFA.

### Proteomic data acquisition

Hydrophilic interaction liquid chromatography fractions were analyzed using LC-MS/MS with a Q-Exactive HF instrument operated in data-dependent mode. Peptides were separated using an UltiMate 3000 RSLCnano system (DIONEX). The capillary column was 30 cm long with an inner diameter of 100 μm, packed with Reprosil Pur C18AQ 3 μm resin. Data acquisition was performed with Xcalibur software (https://www.thermofisher.com) from Thermo Fisher Scientific. Survey scans were conducted in the Orbitrap mass analyzer, covering the mass range of 380 to 2000 *m/z*, with a mass resolution of 60,000 (at *m/z* 200). Tandem mass spectrometry analysis was performed by selecting the most abundant ions with a charge state of 2, 3, or 4 within an isolation window of 2.0 *m/z*. The selected ions were fragmented using higher-energy collisional dissociation with a normalized collision energy of 28, and the tandem mass spectra were acquired in the Orbitrap mass analyzer with a mass resolution of 15,000 (at *m/z* 200).

For the Pre-B and HCT116 phosphoproteomic experiments, the UniProt databases for mouse and human, respectively, were utilized. Peptide identification and quantification were processed using the trans proteomic pipeline tools (55). The search engine employed was Comet (v. 2019.01.1, https://uwpr.github.io/Comet/) (56). Search parameters allowed for semitryptic peptides and included a precursor match tolerance of 15 ppm, differential mass modification of 79.966331 Da for phosphorylated peptides, and a static modification of 57.021465 Da for alkylated cysteine residues. After the searches, peptides were scored using the PeptideProphet algorithm, and SILAC ratios were calculated using XPRESS (http://tools.proteomecenter.org/XPRESS.php). Resultant datasets were filtered using the following parameters: minimum PeptideProphet probability of 0.9, minimum peptide length of 7 amino acid residues, accurate mass binning, and restriction to +2, +3, and +4 ion charge states. Phosphorylated peptides were further evaluated using PTMProphet to obtain a localization score for the modification.

### Phosphorylation motif analysis

To generate sequence logo diagrams, we selected sites within the mouse phosphoproteomes that exhibited at least a 2-fold change upon treatment with IR and/or kinase inhibitors. The phosphorylated residue was fixed as serine for the analyses, as it represented more than 80% of motifs in all phosphoproteomes. These sites were used as foreground data against a background dataset comprising of 9529 unique phosphorylated serine-centered 11-residue sequences, obtained from the Bowtie-filtered IR-treated phosphoproteome (including unregulated and regulated sites). The logo diagrams were generated using the pLogo webserver (40) [https://plogo.uconn.edu]. For motif enrichment analysis of combined +1 and +2 positions, we considered individual P and Q residues, as well as amino acid groups: bulky hydrophobic (ψ – F/I/L/V), acidic (D/E), basic (R/K), phospho-acceptor (S/T/Y), and other (A/G/C/M/N/W/H). This resulted in 49 (7 × 7) potential combined motifs. We then assessed the prevalence of each motif within regulated and nonregulated subsets (Fig. S4*B*) across all phosphoproteomes analyzed. This data was used to generate heat maps and calculate enrichments (regulated/nonregulated).

## Data availability

Mass spectrometry data generated from this study has been deposited to the PRIDE database under the identifier: PXD042258 [https://www.ebi.ac.uk/pride/].

## Supporting information

This article contains supporting information (26).

## Conflict of interest

The authors declare that they have no conflicts of interest with the contents of this article.
