# Supplementary material for: In-depth mapping of DNA-PKcs signaling uncovers noncanonical features of its kinase specificity
Source: J Biol Chem. 2024 Jun 28;300(8):107513. doi: 10.1016/j.jbc.2024.107513 (PMC11327452; doi:10.1016/j.jbc.2024.107513)

**SUPPLEMENTARY TABLES AND FIGURES**

**Supp. Table 1.** Phosphoproteomes of IR irradiated mouse Pre-B cells in the absence or presence of DNA-PKcs and ATM inhibitors.

**Supp. Table 2.** Phosphoproteomes of IR irradiated HCT116 cells in the presence of DNA-PKcs inhibitor.

**Supp. Table 3.** Mapping and comparison of IR-induced, DNA-PKcs dependent homologous sites between mouse and human cells.

**Supp. Figure 1. Phosphoproteomics of IR-treated Mouse Pre-B cells.** A) Scatter plot comparing all phosphosites identified in two quantitative phosphoproteomic analyses of cells treated (+IR) and untreated (-IR) with ionizing radiation. The isotope labeling regime (Light/Heavy) is reversed in the two experiments. B) Following Bowtie filtering (Faca *et al*, 2020), the number of high-confidence sites in quadrants Q2/Q4 and center, indicated by light blue dots within the dashed gray lines, decreased from 16,092 to 13,938.

**Supp. Figure 2. Enrichment of IR-induced S/T-Q sites in the group of phosphorylation sites reduced by the co-treatment with DNA-PKcs and ATM inhibitors.** This figure reproduces the scatter plot from Figure 2C, highlighting IR-induced S/T-Q sites that are dependent (in light red) and independent (in blue) on the activities of DNA-PKcs and ATM.

**Supp. Figure 3. DNA-PKcs and ATM regulate the phosphorylation status of various proteins involved in RNA biology.** String analysis performed with the set of IR-induced sites downregulated by the combined treatment with ATM and DNA-PKcs inhibitors.

**Supp. Figure 4. Motif analysis of IR-induced and kinase-dependent phosphorylation sites.** A) Motif consensus analysis of sub-sets of the sites analyzed in Figure 3. Logos were generated with sequences strictly possessing bulk hydrophobic residues at position +1 (top row) or acidic residues (D/E) at position +2 (bottom row). Serine at the phosphorylation site was fixed in the sequence logos. B) Schematic representation of site groupings used for heatmap generation and S/T-Q/ S/T-ψ-D/E motif enrichment calculations. Regulated phosphorylation sites are induced by IR (Log_2_ ratio of +IR/-IR > 1) and kinase-dependent (Log_2_ ratio of -Inhibitors+IR/+Inhibitors+IR > 1). Non-regulated sites are centrally located (-0.5 < Log_2_ ratios < 0.5). C) Heatmaps showing the prevalence of indicated motifs in the group of non-regulated sites. Motifs are categorized based on the residues at the +1 and +2 positions following the phosphorylated residue. Pie charts at the bottom of the heatmaps depict the relative proportions of S/T-Q and S/T-ψ-D/E motifs.

**Supp. Figure 5. Phosphorylation events identified in the *in vitro* kinase assay.** List of proteins and respective peptide sequences included in the chimeric peptide construct. The columns pS and S show the frequency of identification of the phosphorylated peptide post-incubation with DNA-PK for the wild-type motif S/T-ψ-D/E and the positional mutants S/T-A-D/E and S/T-ψ-A. The ratio is presented in the pS/S columns. The row Total represents the sum of all pS and S in each group, as well as the ratio between the total number of phosphorylated and unphosphorylated PSMs. ND – not defined.

**Supp. Figure 6. Phosphoproteomics of IR-treated Human HCT116 cells in the presence or absence of DNA-PKcs inhibitor.** A) Scatter plot comparing all phosphosites identified in two quantitative phosphoproteomes of IR irradiated cells, one set pre-treated with DNA-PKcs inhibitor (+DNA-PKi) and the other untreated (-DNA-PKi). The isotope labeling regime (Light/Heavy) is reversed in the two experiments. B) After Bowtie filtering (Faca *et al*, 2020), the number of high confidence sites in quadrants Q2/Q4 and center, indicated by light blue dots within the dashed gray lines, reduced from 14,559 to 11,438. C) Heatmap of non-regulated sites (see Suppl. Figure 4B), categorized based on the residues at the +1 and +2 positions following the phosphorylated residue. D) Heatmap of regulated sites (see Suppl. Figure 4B) based on the same motif categorization as in panel C. Pie charts on the right of heatmaps depict the relative proportions of S/T-Q and S/T-ψ-D/E motifs. E) Bar charts illustrating the enrichment of S/T-Q and S/T-ψ-D/E motifs as calculated by the ratio of regulated and non-regulated sites. F) Gene ontology analysis showing enriched biological processes and cellular components among IR induced and DNA-PKcs phosphorylation sites.

**Supp. Figure 7. Cross-species proteins with conserved IR-induced and DNA-PKcs-dependent phosphorylation sites.** In the STRING network analysis, lines represent physical and/or functional interactions. Proteins containing the SAP domain are marked with a black circle.

**Supp. Figure 1.**


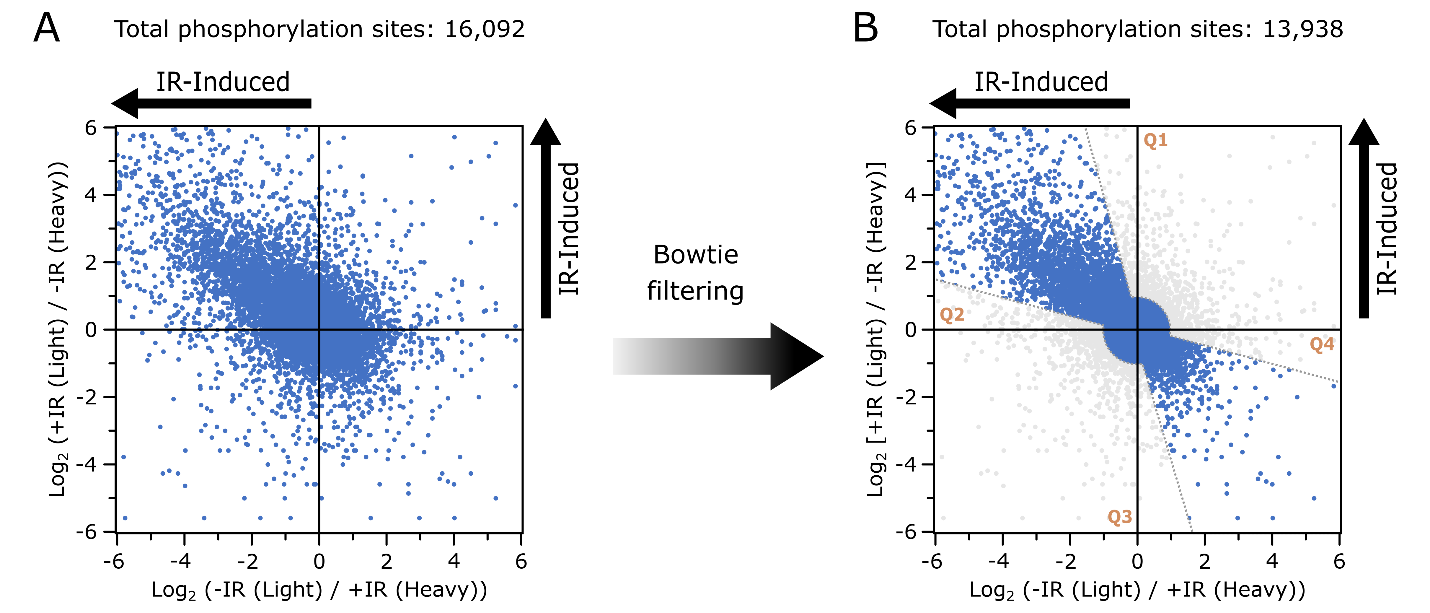


**Supp. Figure 2.**


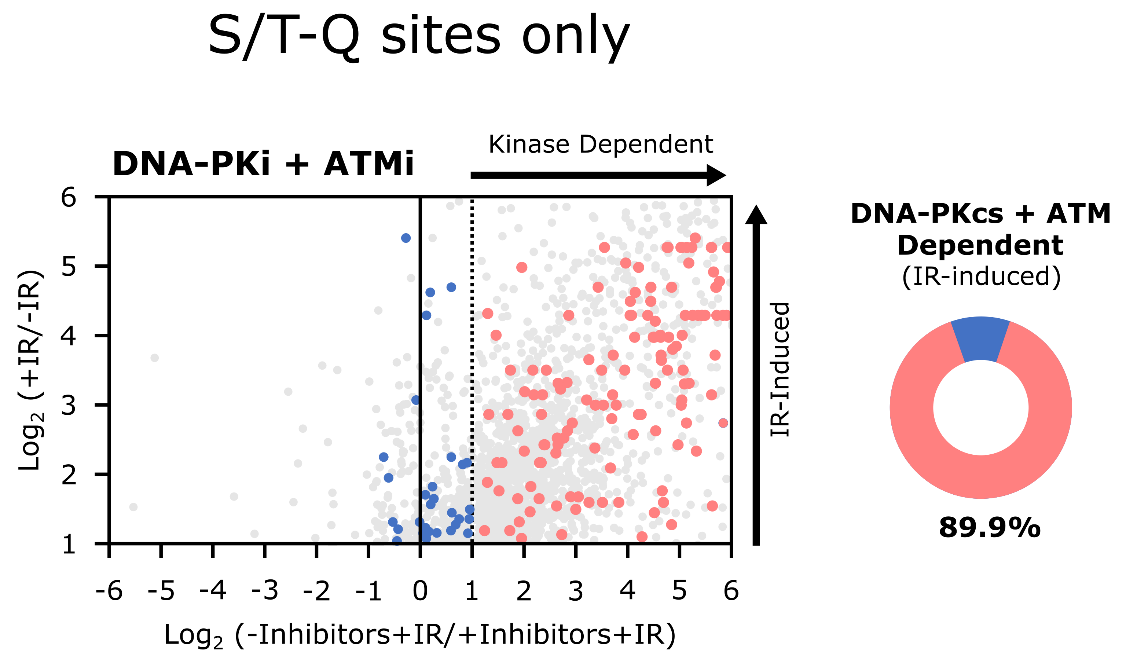


**Supp. Figure 3.**


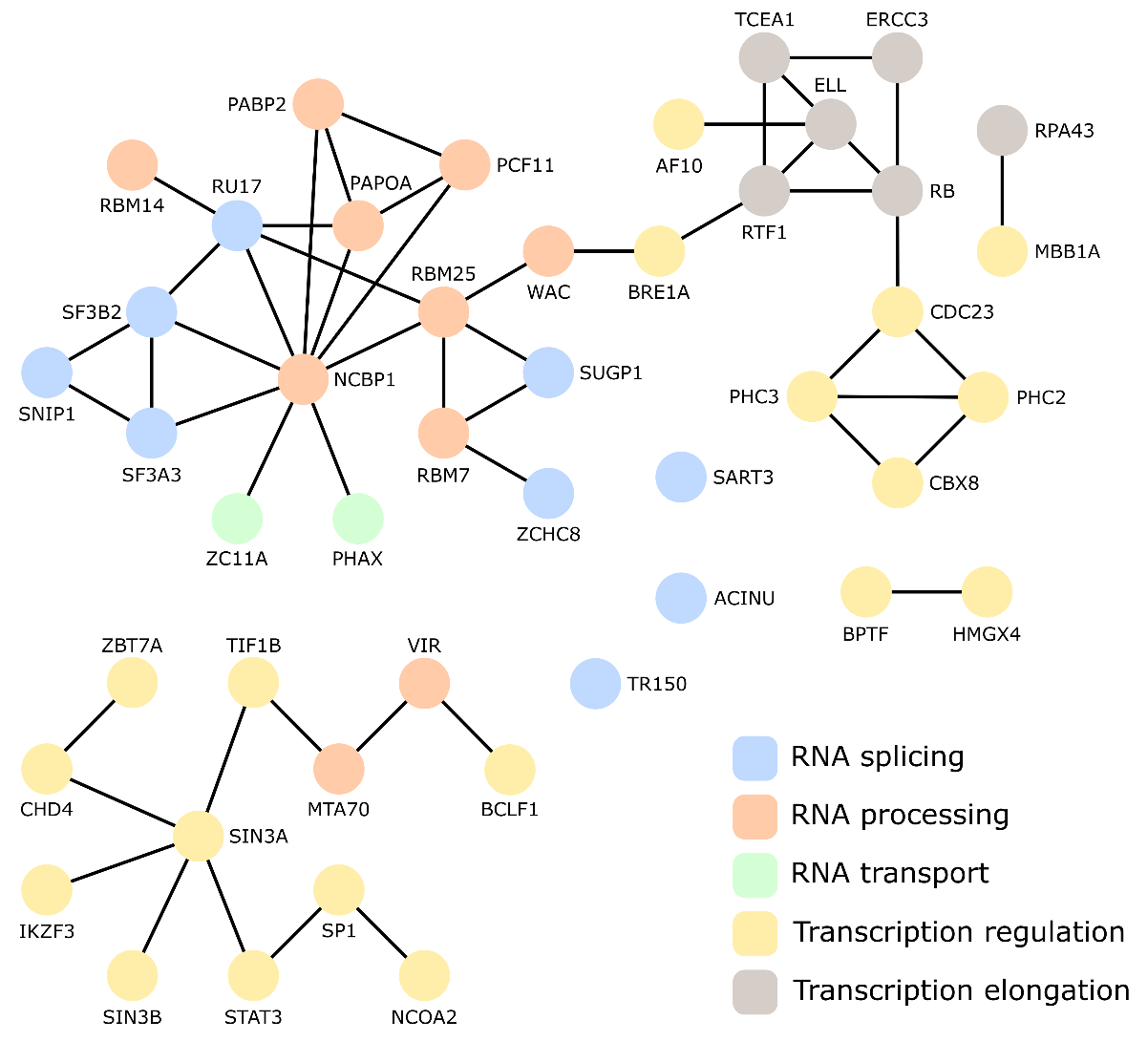


**Supp. Figure 4.**


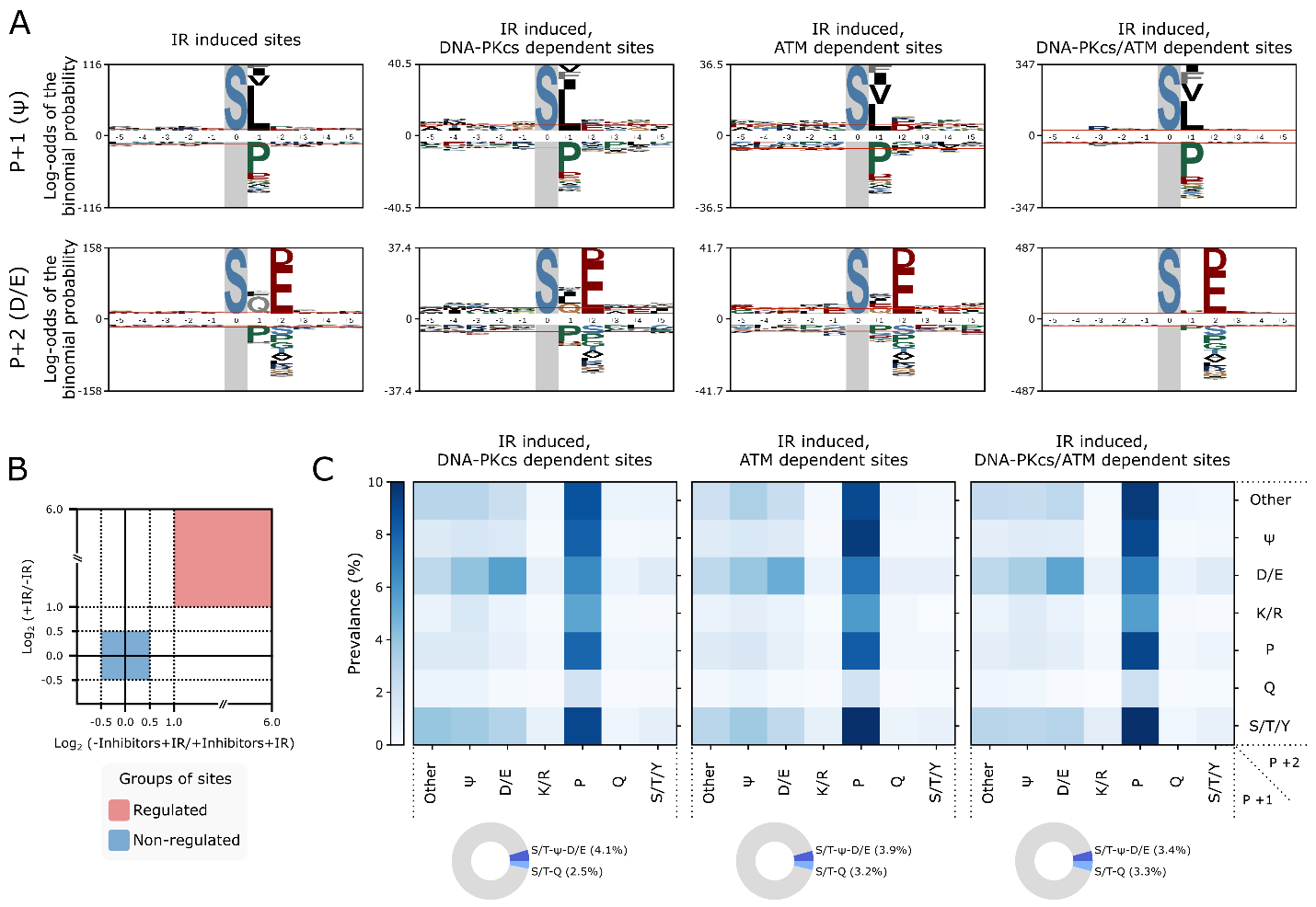


**Supp. Figure 5.**


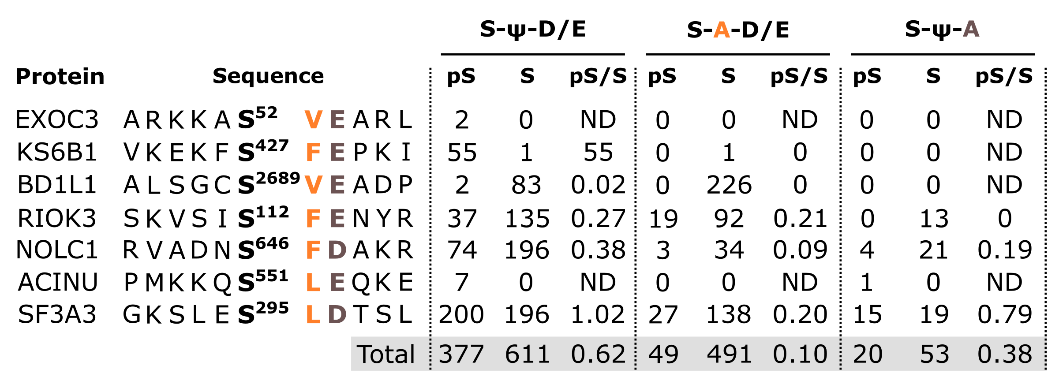


**Supp. Figure 6.**


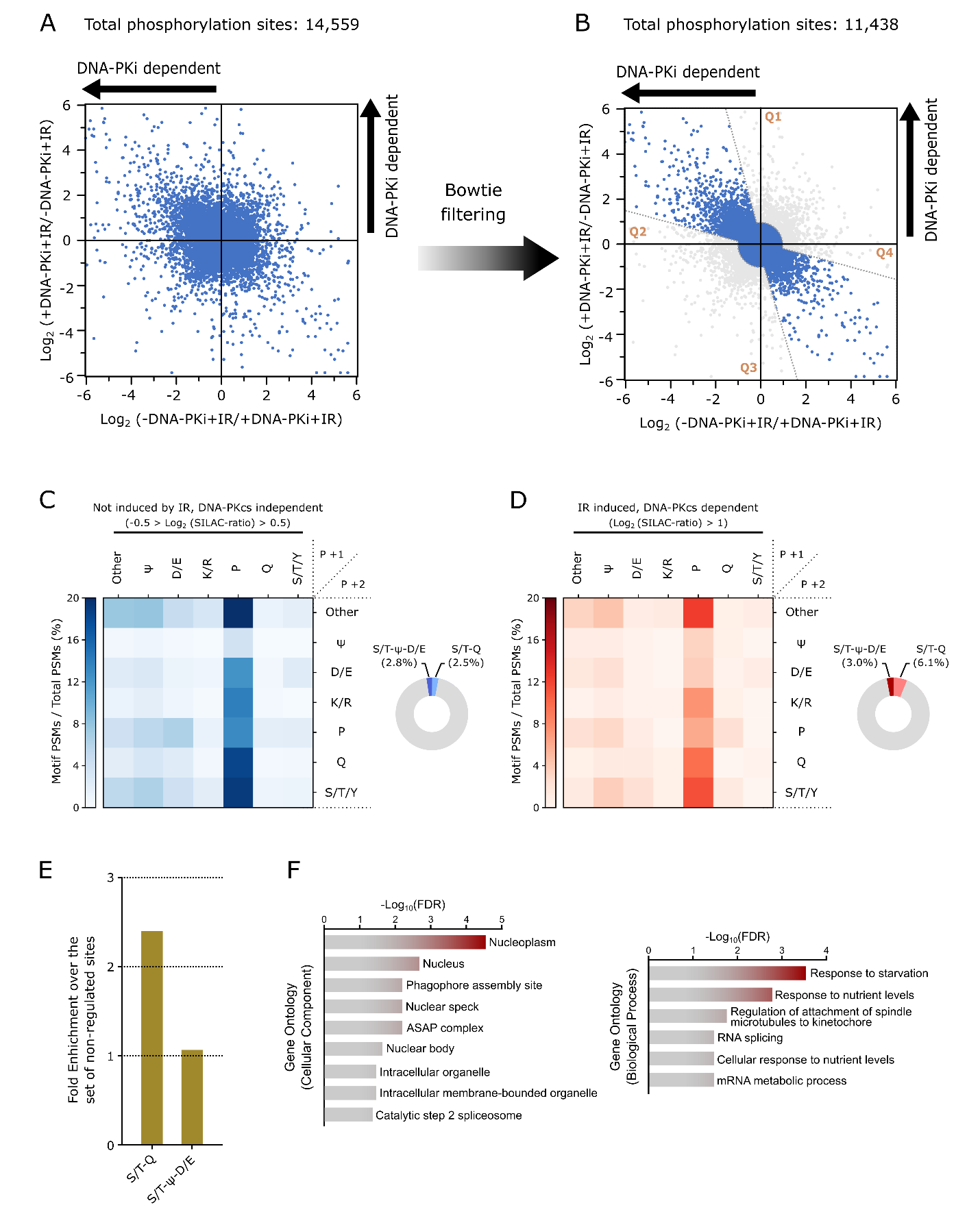


**Supp. Figure 7.**


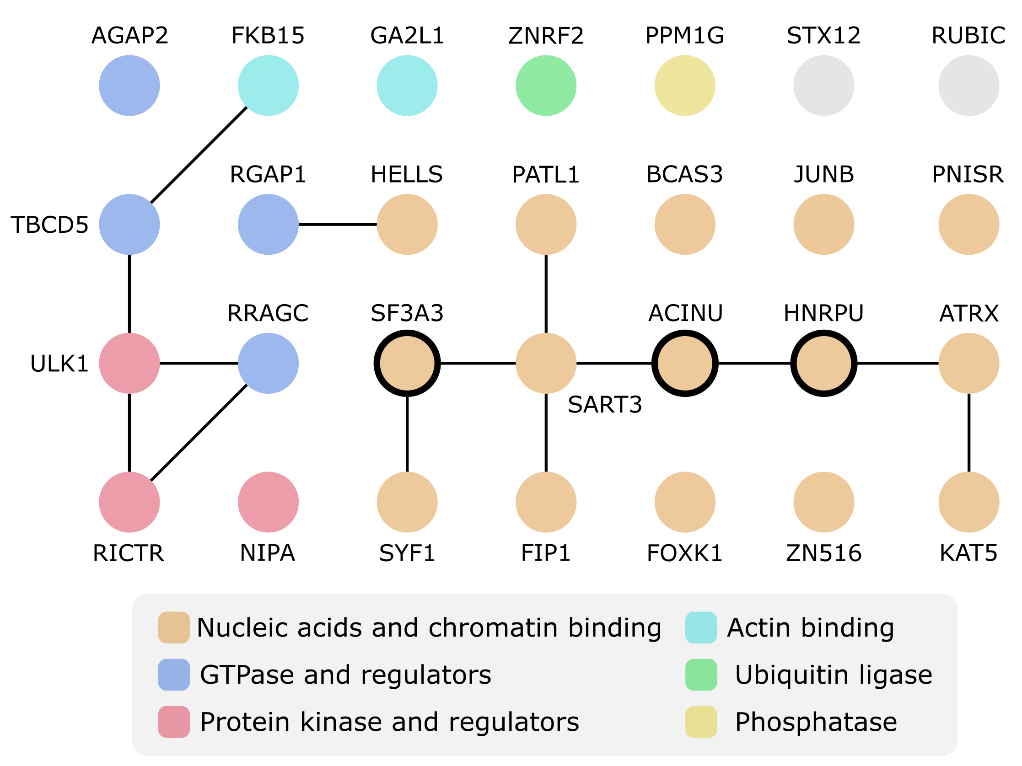

Supplement: Supporting Information [file mmc1.docx]
